# Evaluation of a commercially available organic acid product on body weight loss, carcass yield, and meat quality during preslaughter feed withdrawal in broiler chickens: A poultry welfare and economic perspective^1^

**DOI:** 10.3382/ps.2013-03444

**Published:** 2014-01-21

**Authors:** A. Menconi, V. A. Kuttappan, X. Hernandez-Velasco, T. Urbano, F. Matté, S. Layton, G. Kallapura, J. Latorre, B. E. Morales, O. Prado, J. L. Vicente, J. Barton, R. L. Andreatti Filho, M. Lovato, B. M. Hargis, G. Tellez

**Affiliations:** * Department of Poultry Science, University of Arkansas, Fayetteville 72701; † Facultad de Medicina Veterinaria y Zootecnia, Universidad Nacional Autónoma de México, México D.F. 04510; ‡ Vetanco do Brasil, Rua Raimundo Zanella, 490D Distrito Industrial Flavio Baldissera, Brazil, CEP 89.801-973; § Argentina Vetanco S.A. Chile 33 (B1603CMA) Vicente López, Buenos Aires, Argentina; # Departamento de Producción Agrícola y Animal, Universidad Autónoma Metropolitana, México D.F. 04960; ‖ Facultad de Medicina Veterinaria y Zootecnia, Universidad de Colima, Colima, 28100, México; ¶ Pacific Vet Group-USA Inc., 2135 Creek View Drive Fayetteville, AR 72704; ** Department of Veterinary Clinics, Faculdade de Medicina Veterinária e Zootecnia, Universidade Estadual Paulista, Campus Botucatu, Brazil, 18618-970; †† Depto. de Medicina Veterinária, Centro de Ciencias Rurais, Universidade Federal de Santa Maria, Brazil, 97105-900

**Keywords:** organic acid, chicken, welfare, transportation, meat quality

## Abstract

The effect of a commercial organic acid (OA) product on BW loss (BWL) during feed withdrawal and transportation, carcass yield, and meat quality was evaluated in broiler chickens. Two experiments were conducted in Brazil. Commercial houses were paired as control groups receiving regular water and treated groups receiving OA in the water. Treated birds had a reduction in BWL of 37 g in experiment 1 and 32.2 g in experiment 2. In experiment 2, no differences were observed in carcass yield between groups. Estimation of the cost benefit suggested a 1:16 ratio by using the OA. In experiment 3, conducted in Mexico, significant differences on water consumption, BWL, and meat quality characteristics were observed in chickens that were treated with the OA (*P* < 0.05). These data suggest this OA product may improve animal welfare and economic concerns in the poultry industry by reducing BWL and improving meat quality attributes.

## INTRODUCTION

Global potential for poultry acidifiers, for both feed and water, are on the rise due to higher demand for top quality poultry, which is also true for most of the other animal production operations including swine and cattle (Berkhout, 2009). A global strategic business report on feed acidifiers revealed that the growth potential in the feed acidifiers market is expected to remain robust and expected to rise, mostly attributed to the increasing demand for safe and high-quality pork and poultry meat. Growing awareness and increasing adoption of the use of acidifiers in emerging countries, coupled with escalating demand in the developing world, has expanded the market for these acidifiers (Companies and Markets, 2012).

Europe continues to be the largest regional market with high demand for feed acidifiers in specific, primarily attributed to its large pig and poultry populations, supported largely by legislation that bans the use of antibiotics in feed. Organic acid (**OA**) based feed acidifiers have gained significance due to their high nutritional value and antimicrobial benefits. Major countries dominating the production scene for feed acidifiers include the United States, China, Brazil, Mexico, and Japan, and demand is on the rise in developing regions such as Latin America, Asia-Pacific, and the Middle East (Companies and Markets, 2012).

Most of the research and subsequent applications has been involving feed acidifiers as a preventive or treatment tool for disease management or to improve bird performance. The inclusion of various OA or their salts to diets is shown to improve the growth performance by enhancing the nutrient digestibility and affecting the microbial populations in different parts of the digestive tract (Tung and Pettigrew, 2011). The use of OA or other acidifiers in water management for poultry operations is a subject of much conversation between growers, veterinarians, and live production personnel. Further, research involving the establishment of preferred pH for poultry, its effects on water consumption and eventually poultry welfare, has been limited. Using an OA-based drinking water at critical periods of poultry growth is said to establish and maintain intestinal development by the stability of the intestinal microflora, eventually improving live production performance and cost.

Acidifying drinking water for poultry for the first 7 d of life, when the birds are first placed into the house, is considered critical, because the crop and intestinal microbial morphology would still be under development. Maintenance of low crop pH by the lactic acid bacteria (**LAB**) in newly hatched poults and chicks is critical. The acidified drinking water provides a second layer of protection to the LAB and helps to establish them as a part of the crop's normal ecology. Once the crop's LAB population has been established, the bird will be able to maintain a low crop pH on its own as long as feed is available.

Feed withdrawal (**FW**) for various reasons or when chickens and turkeys are not eating for any reason, leads to an imbalance in the natural population of LAB, leading to an increase in pH, favoring pathogens such as *Salmonella* to multiply in the crop. Preslaughter FW is a method commonly employed to reduce carcass contamination (Corrier et al., 1999; Byrd et al., 2001; Northcutt et al., 2003; Yi et al., 2005). However, carcass shrinkage (carcass dehydration) begins immediately after FW (Benibo and Farr, 1985; Veerkamp, 1986), resulting in recommendations that slaughter take place within 4 to 8 h after FW to minimize losses. Nevertheless, under commercial conditions, this time may be hard to achieve. Consequently, scheduling managers need to consider FW effects on both gut fullness and shrinkage.

In addition to FW, chickens must endure stress during catching, crating transport, and shackling (Gregory, 1994; Petracci et al., 2006). All these factors as well as the total time from FW to slaughter have important implications in welfare of the birds, economics for the poultry industry, and meat quality for consumers (Gregory, 1996; Kannan et al., 1997; den Hertog-Meischke et al., 1997). In poultry and other species, transport-related economic losses are due to mortality, carcass shrinkage, and carcass condemnation (Veerkamp, 1986).

Previously, our laboratory conducted a study in broiler chickens showing that a commercially available water treatment product (Optimizer) significantly reduced carcass condemnation at the processing plant and mortality during transportation, with consistent improvement of average BW at the farm and at the processing plant in broiler chickens (Wolfenden et al., 2007a). In a similar study, the treatment with Optimizer in the drinking water of commercial turkeys during FW showed a significant reduction in the rate of weight loss during transportation and holding at the processing plant in the treated turkeys and improved average BW in treated turkeys during 19 h with an average of 90-g difference (Pixley et al., 2010). Both studies measured BW loss (**BWL**) during holding at the processing plant and the ability to mitigate that loss by treatment with OA before catching. It seems likely that dehydration progressively results in negative welfare for the animal, and the rate of BW change has the potential to be used as a metric in evaluating welfare status of commercial poultry (Warriss et al., 1993; Savenije et al., 2002; Rosenvold and Andersen, 2003; Pixley et al., 2010). Our research has shown the potential to reduce the rate of BWL by administering OA in the drinking water during FW and transportation to the processing plant. In the present study, Optimizer was used in different commercial broiler companies in Brazil and Mexico to evaluate BWL during FW as well as during transportation to the processing plant. Carcass yield and meat quality during preslaughter FW was also assessed, and the implications of poultry welfare and economic results are discussed.

## MATERIALS AND METHODS

### OA

An OA product (Optimizer, Pacific Vet Group-USA Inc., Fayetteville, AR) was used in the drinking water during FW according to manufacturer's directions (4 L of Optimizer/1,000 L of water). This commercial OA product is a combination of 5 different OA (lactic, acetic, tannic, propionic, and caprylic acids) that contains proprietary flavoring agents. This OA product has been shown to reduce *Salmonella* colonization in crop and cecal tonsils without affecting water consumption in chickens (Jarquin et al., 2007; Vicente et al., 2007; Wolfenden et al., 2007b).

### Experimental Design

#### Experiment 1. Effect of the OA Product on BWL During Preslaughter FW and Transportation Under Commercial Conditions in Different States of Brazil.

In experiment 1, 5 trials were conducted in 5 different commercial poultry farms located in 3 different states of Brazil (Table 1). Furthermore, the individual trials were done during the year 2012, with birds having different age and subjected to various FW, transportation, and total fasting periods. In all these trials, houses were designated as control groups receiving regular water and as treated groups receiving OA in the water at a concentration of 4 L/1,000 L of water (vol/vol) according to the manufacturer's directions. In trial 3, 8 commercial chicken houses of market age broiler chickens were paired. In all other trials, 2 commercial chicken houses were paired. A total of 35 birds per house treatment were neck tagged and individually weighed before the FW period and at the time of arrival to the processing facility (after the transportation). The difference between the above 2 BW was taken to determine the BWL under preslaughter commercial conditions. Later, a cost benefit analysis was performed based on the BWL to estimate the economic benefit in administering OA in broiler chickens.

**Table 1.** Experimental designs for experiments 1 and 2 in Brazil

				Time (h)		
Item	State	Date	Age (d)	FW period	Transportation period	Total fasting
Experiment 1						
** **Trial 1	Minas Gerais	March 2012	46	8	1	9
** **Trial 2	Minas Gerais	April 2012	43	8	1	9
** **Trial 3	Paraná	March 2012	42	6	5	11
** **Trial 4	Paraná	May 2012	45	6	5	11
** **Trial 5	Paraná	March 2012	42	7	3	10
** **Trial 6	Mato Grosso do Sul	January 2012	44	3	10	13
Experiment 2						
** **Single trial	Paraná	November 2012	47	8	2	10

#### Experiment 2. Effect of the OA Product on Carcass Yield in Broiler Chickens in Brazil.

This experiment was performed in November 2012 in a poultry farm located in the state of Paraná. Sixteen commercial chicken houses of market age broiler chickens (47 d of age) were paired. In this experiment, 8 houses were designated as control groups receiving regular water and 8 houses as treated groups receiving OA in the water at a concentration of 4 L/1,000 L of water (vol/vol), according to the manufacturer's directions. At each farm, 40 tagged market age broilers per house treatment were individually weighed after FW period and at the time of arrival to the processing facility. Feed withdrawal time was 8 h, and transportation was 2 h, which was a total fasting time of 10 h. Carcass yield was also calculated for the same 40 tagged birds.

#### Experiment 3. Effect of OA on Water Consumption, BWL, and Meat Quality Measurements During 8 h Preslaughter FW in Broiler Chickens from Mexico.

A total of 240 forty-day-old female Cobb 500 broilers were obtained from a commercial farm (Colima, Mexico) and moved to isolation facilities at CVM, University of Colima, Mexico. Broilers were neck tagged and randomly assigned to 8 pens, 4 controls and 4 treated, each pen measuring 3 m^2^ with 30 birds per pen and provided finisher feeder and water ad libitum. Broilers were kept in a temperature-controlled room at 30°C. At 42 d of age, all chickens were weighed and treatment was initiated to 4 pens by adding the OA in the drinking water. Control groups receiving regular water and treated groups receiving OA in the water adjusted to a concentration of 4 L/1,000 L of water (vol/vol) according to the manufacturer's directions. When treatment was initiated, feed was removed from the control and treatment pens and water consumption was monitored in all pens. After 8 h of treatment, all broilers were weighed and final water consumption recorded. Three birds from each pen were humanely killed by cervical dislocation. Breast muscles (pectoralis major) were removed immediately and stored individually in plastic bags at 4°C for 24 h for further analysis of meat quality measurements.

### Meat Quality Measurements

At 24 h postmortem, the breast meat pH was determined on individual fillets according to the method as described by Qiao et al. (2002). The pH was determined using a model pH/ISE meter, calibrated at pH 4.0 and 7.0, and was conducted on the medial bone side as follows: a cut approximately 0.5 cm in length and depth was made in the meat, and a drop of deionized water was placed in the cut to improve contact with the pH probe. The probe was rinsed with deionized water and was dried with a filter between samples, and was cleaned with alcohol after every lot of 3 fillets.

The complete International Commission on Illumination system color profile of lightness (L*), redness (a*), and yellowness (b*) was measured on the cranial and medial surface (bone side) using a reflectance colorimeter (Minolta Chroma Meter CR-10, Minolta, Osaka, Japan), in an area free of obvious color defects (bruises, blood spots, or surface discolorations) at room temperature (25 ± 2°C), immediately after samples were tagged. Measurements were made on the medial surface to avoid breast fillet surface discolorations due to possible over scalding in the plant. Color values were calibrated using a Minolta calibration plate (L* = 60.5, a* = −3.2, and b* = +6.7).

Water-holding capacity (**WHC**) of the breast meat samples were measured according to the method as described by Lu et al. (2006), with some modifications. A 0.3-g sample of breast muscle was pressed onto an oven-dried Whatman 125 mm filter paper at 13,789,500 Pa (2,000 psi) for 1 min. The WHC values were calculated as the ratio of the area of expressed water to the area of the pressed meat sample, measured with a planimeter. Therefore, a lower ratio indicates a greater WHC.

Thawing loss (**TL**) was measured according to Mortensen et al. (2006). Immediately before freezing, samples were weighed. The frozen samples were thawed over a period of 24 h at 4°C and weighed again. The TL was determined as the percentage of BWL after thawing.

Drip loss (**DL**) was conducted according to Berri et al. (2008). The muscle samples were weighed and immediately placed in a plastic bag, hung from a hook, and stored at 4°C for 48 h. After hanging, the sample was wiped with absorbent paper and weighed again. The difference in weight corresponded to the DL and was expressed as the percentage of the initial muscle weight.

For cook loss (**CL**), the individually weighed fillets were placed on stainless steel trays and cooked for 30 min at 98°C in steam. Upon removal from the oven, the fillets were covered with plastic film and allowed to equilibrate to room temperature (25°C). Individual fillets were then reweighed to determine CL.

### Data Analysis

Body weight, carcass yield, and meat quality data collected were subjected to one-way ANOVA using the GLM procedure of SAS, with significance reported at *P* < 0.05, means were further separated using Duncan's multiple range test (2002, SAS Institute Inc., Cary, NC).

### Formulas and Estimated Values

\begin{equation*} {\rm Difference \, in \, BWL/chicken = BWL \, of \, nontreated/BWL\, of\, treated} . \end{equation*}

\begin{equation*} {\rm Reduction\, in\, BWL\, of\, total\, chickens = (total\, treated\, chickens) } \times {\rm (difference\, in\, BWL\, of\, treated\, chickens)} . \end{equation*}

\begin{equation*} {\rm Value\, of\, treatment\, for\, total\, chickens = (weight\, gain\, of\, total\, chickens) } \times {\rm [value\, of\, the\, meat/kg\, (estimated\, at\, US\, $\, 1}{\rm .44/kg)]} . \end{equation*}

\begin{equation*} {\rm Total\, water\, consumption = (water\, consumption/chicken) } \times {\rm (total\, treated\, chickens)} . \end{equation*}

\begin{equation*} {\rm Total\, cost\, of\, Optimizer = [cost\, of\, Optimizer/L\, (estimated\, at\, US\, \$ \,4}{\rm .16/L)] } \times {\rm (L\, of\, Optimizer\, used)} . \end{equation*}

\begin{equation*} {\rm Benefit\, to\, cost\, ratio = value\, of\, treatment/Optimizer\, product\, cost\, (expressed\, as\, cost:benefit)} . \end{equation*}

## RESULTS

The results of the effect of the OA on BWL during preslaughter FW and transportation under commercial conditions in different states of Brazil from experiment 1 are summarized in Table 2. In trials 1, 2, and 5, a significant reduction in BWL was observed in the chickens treated with OA compared with control birds (*P* < 0.05), and numerical reduction in trials 3 and 4. Overall average from all 5 trials, treated birds had a reduction in BWL of 37 g compared with control nontreated chickens. Similar results have been reported previously (Wolfenden et al., 2007a; Pixley et al., 2010).

**Table 2.** Effect of the organic acid product (OA) on BW loss during preslaughter feed withdrawal and transportation under commercial conditions in different states of Brazil from experiment 1^1^

Trial	Treatment	Initial BW (g)	BW after transportation (g)	BW change (g)
Trial 1	Control	2,761 ± 43^a^	2,578 ± 50^a^	−183 ± 23^b^
	OA	2,796 ± 57^a^	2,682 ± 57^a^	−114 ± 12^a^
** **BW difference				69
Trial 2	Control	2,674 ± 39^b^	2,533 ± 37^b^	−141 ± 12^b^
	OA	2,797 ± 31^a^	2,702 ± 31^a^	−95 ± 6^a^
** **BW difference				46
Trial 3	Control	3,069 ± 68^a^	2,995 ± 74^a^	−74 ± 37^a^
	OA	3,044 ± 65^a^	2,992 ± 54^a^	−52 ± 12^a^
** **BW difference				22
Trial 4	Control	3,202 ± 64^a^	3,158 ± 65^a^	−44 ± 82^a^
	OA	3,183 ± 63^a^	3,159 ± 62^a^	−24 ± 79^a^
** **BW difference				20
Trial 5	Control	2,793 ± 70^a^	2,724 ± 64^a^	−69 ± 7^b^
	OA	2,757 ± 57^a^	2,736 ± 60^a^	−21 ± 5^a^
** **BW difference				48
Average BW difference				37

^a,b^Different superscripts within rows of each trial indicate significant differences (*P* < 0.05), n = 35 birds.

^1^BW data are expressed as means ± SE.

The results of the effect of the OA product on carcass yield in broiler chickens in Brazil from experiment 2 are summarized in Table 3. In this experiment, a numerical reduction of 32.2 g of BWL was observed in treated chickens compared with control nontreated chickens. Remarkably, no differences were observed in carcass weight and carcass yield between treated and control chickens. Carcass yield (%) in control chickens was 76 versus 75.9% in OA-treated chickens.

**Table 3.** Effect of the organic acid (OA) product on BW and carcass yield in broiler chickens in Brazil from experiment 2^1^

Item	Control	OA
Initial BW (g)	3,040 ± 31^a^	2,990 ± 33^a^
BW after transportation (g)	3,004 ± 31^a^	2,986 ± 32^a^
BW loss (g)	36.8 ± 37^a^	4.8 ± 32^a^
Difference (g)	32.2	
Carcass weight (g)	2,967 ± 37^a^	2,981 ± 38^a^
Carcass yield (%)	76.0	75.9
Difference (%)	0.1	

^a^*P* > 0.05, n = 40 birds.

^1^BW data are expressed as mean ± SE.

The economic analysis from experiments 1 and 2 on chickens treated with the OA product is shown in Table 4. From this analysis, the reduction in BWL when converted to a cost:benefit ratio suggested that for every $1 US spent with this OA product, producers were able to recover on average $16 US.

**Table 4.** Cost:benefit ratio of organic acid (OA) product from experiments 1 and 2

Trial and state	Cost:benefit ratio^1^ ($, US)
Experiment 1	
** **Trial 1, Minas Gerais	1:15
** **Trial 2, Minas Gerais	1:12
** **Trial 3, Paraná	1:14
** **Trial 4, Paraná	1:17
** **Trial 5, Mato Grosso do Sul	1:17
Experiment 2	
** **Trial 1, Paraná	1:20
Overall average	1:16

^1^Estimated according to the numbers of broiler chickens/house treated with OA.

Table 5 summarizes the effect of OA on BWL in broiler chickens during 10 h preslaughter FW, from experiment 3. As it has been shown previously (Jarquin et al., 2007; Vicente et al., 2007), unlike treatment with lactic or formic acid (Byrd et al., 2001), the OA treatment used in the present study showed a significant increase in water consumption that was associated with a significant minor BWL after 10 h of FW compared with nontreated birds. Table 6 summarizes the results of OA on raw breast meat color, chemical composition, pH, moisture, and water-holding characteristics in broiler chickens after 10 h of FW from broilers in experiment 3. A significant increase in lightness, redness, DL, and CL with a significant reduction on meat pH, moisture, and WHC was observed in nontreated chickens compared with OA-treated chickens.

**Table 5.** Effect of organic acids on BW loss in broiler chickens during 10 h preslaughter feed withdrawal in Mexico from experiment 3^1^

Item	Control	Optimizer
Water consumption (mL/bird)	38 ± 3^b^	54 ± 5^a^
Initial BW (g)	2,830 ± 50^a^	2,798 ± 43^a^
BW after 10 h of feed withdrawal (g)	2,657 ± 35^b^	2,672 ± 54^a^
BW loss (g)	176 ± 45^a^	128 ± 57^b^
Difference (g)	48	37

^a,b^Treatments values within rows with no common superscript differ significantly (*P* < 0.05).

^1^Data are expressed as mean ± SE. Each group with 4 replicates of n = 30 each replicate.

**Table 6.** Effect of the organic acids on raw breast meat color, pH, moisture, and water-holding characteristics in broiler chickens during preslaughter feed withdrawal in Mexico from experiment 3^1^

	Breast meat characteristic					Water-holding characteristic (%)			
Treatment	Lightness (L*)	Redness (a*)	Yellowness (b*)	pH	Moisture	Water-holding capacity	Thawing loss	Drip loss	Cook loss
Control	40.4 ± 0.7^a^	5.6 ± 0.1^a^	10.3 ± 0.9^a^	5.4 ± 0.2^a^	69.6 ± 0.7^b^	65.4 ± 0.3^b^	5.4 ± 0.5^a^	6.8 ± 0.2^a^	36.1 ± 0.7^a^
Organic acids	37.4 ± 0.1^b^	4.7 ± 0.3^b^	11.4 ± 0.5^a^	6.0 ± 0.1^b^	72.0 ± 0.3^a^	68.2 ± 0.5^a^	5.0 ± 0.6^a^	4.1 ± 0.5^b^	33.4 ± 0.9^b^

^a,b^Treatment values within columns with no common superscript differ significantly (*P* < 0.05), n = 12 birds.

^1^Data expressed as means ± SE.

## DISCUSSION

Prior to slaughter, broiler chickens are exposed to many handlings and conditions such as FW, catching, crating, transport, and shackling that have a profound impact in their welfare (Ali et al., 1999; Akşit et al., 2006; Petracci et al., 2006; Vanderhasselt et al., 2013). Under those stressful circumstances, energy reserves of the birds can be severely affected, modifying their metabolic state at slaughter, which has a negative effect in the final meat quality for the consumers (Gregory, 1996; Kannan et al., 1997; Ali et al., 1999; Petracci et al., 2001).

Feed withdrawal is a common practice that is intended to reduce fecal contamination of carcasses; however, during transport and lairage birds also experience water withdrawal (Corrier et al., 1999; Northcutt et al., 2003). All things considered (FW, crating time, transport, and lairage) could add a minimal of 9 h of feed deprivation, although infrequently, much longer times have been reported (Warriss et al., 1990), which will lead to significant carcass shrinkage (Veerkamp, 1986). Nevertheless, lack of feed and water has been reported to reduce glycogen levels in liver following as little as 3 h of FW (Warriss et al., 1988), which has also correlated with a significant decrease in postmortem liver pH (Warriss et al., 1993).

In the present study, the use of the OA product showed a significant or numerical reduction in BWL during FW period and transportation (Tables 2, 3, and 5). The combination of the OA used in the Optimizer could have helped to improve the weight loss (Jarquin et al., 2007), even though the use of individual OA alone did not produce such an effect (Byrd et al., 2001). This implies that Optimizer had a benefit from the bird welfare point of view that it did not cause much dehydration, in addition to its documented *Salmonella*-recovery reductions in market age broilers when administered during the preslaughter FW period (Jarquin et al., 2007; Wolfenden et al., 2007a).

The significant increase in lightness and redness, increase drip and cooking loss as well as significant reduction on meat pH, moisture, and WHC observed in control nontreated chickens compared with OA-treated chickens (Table 6), suggest that the increased water consumption observed in previous studies (Jarquin et al., 2007; Vicente et al., 2007; Wolfenden et al., 2007a) and confirmed in this study (Table 5) may improve the physiological hydration state of the birds. From the results of carcass yield in experiment 2, where percentage carcass yield had a slight difference of 0.1% between treated and control group (yet a numerical difference in BWL of 32.2 g) and meat quality results observed in experiment 3 (Tables 3 and 6), we can infer that, perhaps, the increase water consumption induced by this OA product is retained in the muscle and is not released in feces/urine or when blood and viscera are removed. This observation was supported in the present study by a consistent significant or numerical improvement in BWL (Tables 2, 3, and 5).

Several investigators have shown that the distribution and mobility of water in muscle (myowater) and meat have a profound influence on essential meat quality (Benibo and Farr, 1985; Castellini et al., 2002; Bertram et al., 2003; Pearce et al., 2011). During the conversion of the living muscle to meat and during aging, the myowater content, location, and mobility will change as a function of numerous mutual interacting factors of both ante and postmortem biochemistry (Ali et al., 1999; Bertram et al., 2003; Bond et al., 2004; Akşit et al., 2006). After death, oxygen supply is stopped, and energy has to be generated under anaerobic conditions resulting in accumulation of lactic acid, which decrease the pH of the muscle and affect the color of the meat and WHC due to protein breakdown (Warriss and Brown, 1987). When this anaerobic energy supply fails, rigor mortis appears (Maribo et al., 1998), which is directly correlated with the glycogen reserves of the birds and the metabolic state of the muscle before slaughter. The cessation of postmortem energy production in chickens has been reported to happen within 6 h of FW (Grey et al., 1974).

The WHC of chicken meat products is related to final carcass yield which affect economics and eating quality such as juiciness and tenderness (Zamorano and Gambaruto, 1997; Dai et al., 2009). Several ante mortem and postmortem factors have been reported to affect the conversion of living muscle to meat as well as the location and content of the myowater (Pearce et al., 2011). Therefore, any loss of water reduces the weight of the product, which contributes to financial loss through loss of salable product. Most of the water in the muscle fibers is present in the myofibrils, which represent about 80% of the muscle volume (Cheng and Sun, 2008). When the muscles are cut, a red fluid, called drip, exudes from the cut surfaces. This solution consists primarily of myoglobin and glycolytic enzymes (Cavitt and Sams, 2003). Excessive DL not only affects the final yield of the carcass, it also affects the protein concentration of the meat and represent a safety concern because this fluid is an excellent nutrient broth for spoiling and pathogenic bacteria (den Hertog-Meischke et al., 1997; Castellini et al., 2002; Pedersen et al., 2003; Northcutt et al., 2003). This is the first report that demonstrate that this OA induced increased water consumption and reduction of BWL during FW, catching, crating, transport, and shackling of poultry, which are associated with a positive improvement of meat quality attributes, such as color of the meat, pH, moisture, and overall water holding characteristics (higher WHC and lower DL and CL). Furthermore, the reduction in BWL when converted to a cost:benefit ratio suggested that for every US dollar spent on this OA product, producers may be able to recover on average $16 US.
